# Dataset of cortical activity recorded with high spatial resolution from anesthetized rats

**DOI:** 10.1038/s41597-021-00970-3

**Published:** 2021-07-15

**Authors:** Csaba Horváth, Lili Fanni Tóth, István Ulbert, Richárd Fiáth

**Affiliations:** 1 Institute of Cognitive Neuroscience and Psychology, Research Centre for Natural Sciences, Eötvös Loránd Research Network, Budapest, Hungary; 2 School of Ph.D. Studies, Semmelweis University, Budapest, Hungary; 3 Faculty of Information Technology and Bionics, Pázmány Péter Catholic University, Budapest, Hungary

**Keywords:** Neural circuits, Extracellular recording, Slow-wave sleep, Action potential generation

## Abstract

Publicly available neural recordings obtained with high spatial resolution are scarce. Here, we present an electrophysiological dataset recorded from the neocortex of twenty rats anesthetized with ketamine/xylazine. The wideband, spontaneous recordings were acquired with a single-shank silicon-based probe having 128 densely-packed recording sites arranged in a 32 × 4 array. The dataset contains the activity of a total of 7126 sorted single units extracted from all layers of the cortex. Here, we share raw neural recordings, as well as spike times, extracellular spike waveforms and several properties of units packaged in a standardized electrophysiological data format. For technical validation of our dataset, we provide the distributions of derived single unit properties along with various spike sorting quality metrics. This large collection of *in vivo* data enables the investigation of the high-resolution electrical footprint of cortical neurons which in turn may aid their electrophysiology-based classification. Furthermore, the dataset might be used to study the laminar-specific neuronal activity during slow oscillation, a brain rhythm strongly involved in neural mechanisms underlying memory consolidation and sleep.

## Abstract (summary)

**Table:** 

Measurement(s)	cortical electrical activity
Technology Type(s)	electrophysiological method
Sample Characteristic - Organism	Rattus norvegicus

Machine-accessible metadata file describing the reported data: 10.6084/m9.figshare.14555421

## Background & Summary

Neurons transmit information to their postsynaptic peers via short electrical impulses called action potentials or spikes. This form of communication can be detected and monitored using extracellular electrophysiological methods where single or multiple neural probes with thin implantable shanks are inserted into the brain tissue near the neurons of interest. State-of-the-art extracellular probes contain hundreds of miniature recording sites on their shanks allowing to monitor and record the activity of hundreds or even thousands of neurons simultaneously^1–5^. Although electrophysiological techniques have submillisecond temporal resolution, the spatial resolution of most neural probes is poor because of technical limitations and spatial constraints^6^. Recent developments in microfabrication technology, however, made it possible to construct high-density probes comprising a high number of small, closely packed recording sites which allow to record spiking activity in the brain with a significantly enhanced spatial resolution^1,7–12^. Benefits of spatially dense recordings acquired with these improved devices include, for example, a more detailed spatiotemporal profile of action potential waveforms of individual neurons (i.e., the spike waveform of a single unit is detected by multiple adjacent sites simultaneously), the potential for the correction of electrode drift or the tracking of the position of single units^7,13^. A finer, more detailed electrical footprint of spikes may be exploited to increase the accuracy and reliability of spike sorting algorithms, to locate the soma position of neurons more precisely or to distinguish various neuron types within or between brain regions with a greater reliability^14,15^. Although high-density devices (e.g., Neuropixels probes) are already in use in many neuroscience laboratories, publicly available high-resolution electrophysiological recordings are still scarce^3,12,16–18^.

Here, we provide a large electrophysiological dataset (n = 109 recordings, ~0.9 TB overall size) collected from the neocortex of twenty anesthetized rats with a 128-site silicon probe providing high spatial resolution^7^. In addition to making the continuous raw and wideband recordings publicly available, we share the results of spike sorting, including the spike times, multichannel spike waveforms and several spatiotemporal properties of single units. Along with the neural signals, various sorting quality metrics are also published to aid potential future users in selecting single units with a quality appropriate for their research. To make the dataset available for a wider user base, it is packaged in the Neurodata Without Borders (NWB) format, a standardized data format used in the field of electrophysiology^19–22^. A part of this dataset was used in one of our previous studies, where we compared the recording performance of sites located at the edge and in the middle of the silicon shank of various probe types^23^.

This high-density cortical dataset might be used, for example, to compare and verify the results of computational models of extracellular action potentials of different neuron types with *in vivo* recorded spike waveforms^24^. Also, these recordings provide the opportunity to construct hybrid ground truth data which can be applied to test and validate novel spike sorting algorithms or to improve existing methods^25^. Furthermore, the high number of single units available in our dataset allows to examine whether there might be significant differences in the spatial features of spike waveforms of different neuron types, and whether these differences might facilitate neuron classification or identification^14,15^. Finally, this dataset can be reused to study the spatiotemporal patterns of the cortical activity (e.g., between cortical layers) during the slow oscillation (also referred to as slow-wave activity), a brain rhythm which emerges during slow-wave sleep and also under ketamine/xylazine anesthesia, and has a key role in the consolidation of memories acquired during wakefulness^9,26,27^.

## Methods

### High-density silicon probe used for electrophysiological recordings

**Fig. 1:**
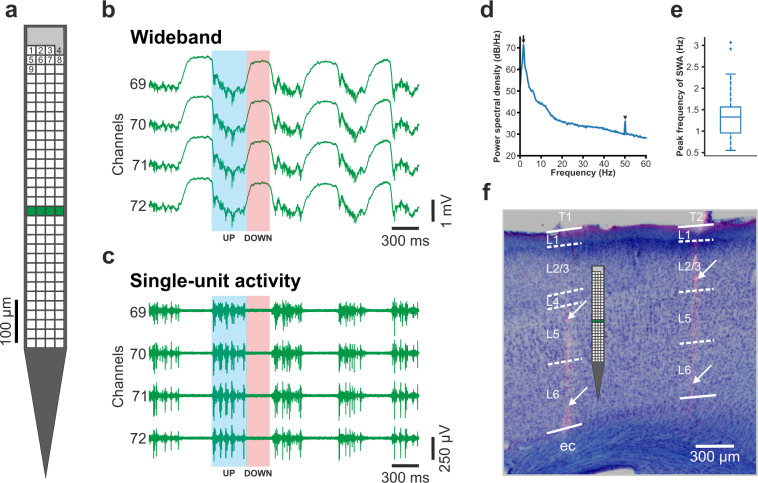
Cortical slow-wave activity (SWA) recorded with high spatial resolution. (**a**) Schematic of the 128-site silicon probe used to collect the dataset. Recording sites (white squares) are arranged in a 32 × 4 dense array. The site located in the upper right corner of the array (with gray color) has a larger area and might be used as an internal reference electrode. The recording site – channel mapping is indicated by the numbering located at the top of the array. (**b**,**c**) Three-second-long wideband (0.1–7500 Hz) and highpass-filtered (500 Hz; single-unit activity) layer 5 activity in the cortex recorded on four channels (green sites on panel a). Under ketamine/xylazine anesthesia, SWA is the dominant brain rhythm emerging in the cortex. During SWA, two phases alternate: up-states (high spiking activity; example shown with the blue shaded area; UP) and down-states (cessation of spiking activity; example indicated with the red shaded area; DOWN). (**d**) Power spectrum of a recording from deep cortical layers demonstrating the characteristic peak frequency of the SWA at ~1.5 Hz (black arrow). The arrowhead indicates the degree of contamination by the power line (50 Hz) noise. (**e**) Boxplot showing the distribution of SWA peak frequencies of all recordings (n = 109). The central mark indicates the median, the bottom and top edges of the box mark the 25th and 75th percentiles, respectively. The whiskers extend to the most extreme data points not considered outliers. Outliers are plotted individually using the ‘+’ symbol. The features of boxplots are the same on following figures. (**f**) The recording positions of the silicon probe (tracks) were verified by examining fluorescent marks of DiI in the cortical brain sections (white arrows). Nissl-staining was used to determine the borders of cortical layers. The probe schematic shows the estimated cortical depth from where traces on panel b and c were acquired. The probe track on the left (T1) is located in the primary somatosensory cortex, while the track on the right (T2) is in the motor cortex. L, layer; ec, external capsule.

To record the cortical electrical activity, we used a single shank, multisite silicon-based probe with 128 closely spaced titanium nitride electrodes^7^ (Fig. 1a). This custom-designed silicon probe was fabricated at the Interuniversity Microelectronics Center (IMEC; Leuven, Belgium) during the NeuroSeeker project (www.neuroseeker.eu) and is not commercially available. This probe type has an 8 mm long implantable shank with a cross-section of 100 μm × 50 μm (width (W) × thickness(T)) and with a chisel-shaped tip. The square-shaped recording sites located on the shank have 20 μm side lengths (site area, 400 μm^2^), and the spacing between the edges of the sites is 2.5 μm (corresponding to a center-to-center site distance of 22.5 μm). The recording sites form a 32 × 4 dense array with equidistant spacing between the sites. All microelectrodes have a low impedance of about 50 kΩ at 1 kHz^7^, except for one site which has a larger area (and thus a lower impedance of ~10 kΩ). This site, which is located at the top right corner of the array, can be used as an internal reference electrode, however, we used it as a conventional recording site during the acquisition of our cortical recordings (Fig. 1a). The distance between the bottom row of recording sites and the probe tip is 300 μm, and the dense array of microelectrodes covers an area of 87.5 μm × 717.5 μm (horizontal × vertical). This type of silicon probe was used in multiple studies, it provided high-quality neural recordings in acute experiments both from rodents and cats^7,23,28–31^. To examine the long-term performance of the device, it was also chronically implanted in monkeys^32^.

### Animal surgery

All experiments were performed according to the EC Council Directive of September 22, 2010 (2010/63/EU), and all procedures were reviewed and approved by the Animal Care Committee of the Research Centre for Natural Sciences and by the National Food Chain Safety Office of Hungary (license number: PEI/001/2290-11/2015). Acute *in vivo* experiments were carried out similarly as described in our earlier studies^7,23,28^. In short, Wistar rats (n = 20; weight, 315.5 ± 59.6 g; 11 females) were anesthetized by an intramuscular or intraperitoneal injection of a mixture of ketamine (75 mg/kg of body weight) and xylazine (10 mg/kg of body weight). To maintain the depth of anesthesia during surgery and recordings, and to sustain the ongoing cortical slow-wave activity^26,33^, supplementary intramuscular injections were given to the rats regularly using the same anesthetic cocktail. A homeothermic heating pad connected to a temperature controller (Supertech, Pécs, Hungary) was used to maintain their physiological body temperature. The deeply anesthetized rats were placed in a stereotaxic frame (David Kopf Instruments, Tujunga, CA, USA), then we removed the skin and the connective tissue from the top of their skull. Following that, a square-shaped cranial window with the size of about 3 × 3 mm^2^ was drilled over the neocortical areas of interest (trunk or hindlimb region of the primary somatosensory cortex [S1Tr and S1HL, respectively]; primary or secondary motor cortex [M1 or M2, respectively] or the parietal association cortex [PtA]; Table 1). The approximate stereotaxic coordinates of the target sites (with respect to the bregma^34^) are listed in Table 1. Then, to avoid excessive brain dimpling during the insertion of the single-shank silicon probe, the dura mater was carefully pierced above the insertion sites using a sharp 34-gauge needle.

**Table 1:** Animal characteristics, targeted cortical areas and corresponding stereotaxic coordinates.

Animal ID	Weight (g)	Sex	CoA (I1)	AP (I1)	ML (I1)	CoA (I2)	AP (I2)	ML (I3)
Rat01	320	F	S1HL	−2,28	2,44	M1	−1,92	1,39
Rat02	300	F	PtA	−3,12	2,41	M2	−3,24	1,57
Rat03	340	F	S1HL	−1,95	2,44	M1	−1,95	1,87
Rat04	390	F	PtA	−3,60	2,57	S1Tr	−2,40	2,40
Rat05	390	M	PtA	−3,25	2,36	PtA	−3,50	1,45
Rat06	400	M	S1Tr	−2,75	1,99	M1	−2,70	1,60
Rat07	320	M	S1HL	−2,16	2,63	—	—	—
Rat08	320	F	PtA	−3,48	2,96	PtA	−3,36	2,00
Rat09	200	F	M1	−3,12	2,14	M2	−3,12	1,66
Rat10	280	F	PtA	−3,24	2,04	M2	−3,24	1,50
Rat11	240	F	PtA	−3,48	2,32	PtA	−3,48	1,80
Rat12	310	M	S1Tr	−2,50	2,49	M1	−2,50	1,76
Rat13	390	M	S1Tr	−2,64	2,35	M1	−2,64	1,59
Rat14	260	F	S1Tr	−2,80	2,10	M1	−2,90	1,85
Rat15	270	M	PtA	−3,40	2,28	PtA	−3,40	1,31
Rat16	280	F	S1Tr	−2,50	2,68	M1	−2,60	1,66
Rat17	390	M	S1Tr	−2,70	2,49	M1	−2,90	1,97
Rat18	380	M	S1HL	−2,35	1,95	M1	−2,35	1,52
Rat19	290	M	S1Tr	−2,90	2,67	M1	−2,90	1,85
Rat20	240	F	PtA	−3,20	2,25	M1	−3,00	1,71

CoA, Cortical Area; I1, first insertion site; I2, second insertion site; M1, primary motor cortex; M2 secondary motor cortex; PtA, parietal association cortex; S1HL, hindlimb region of the primary somatosensory cortex; S1Tr, trunk region of the primary somatosensory cortex; AP, anterior-posterior; ML, medial-lateral.

**Fig. 2:**
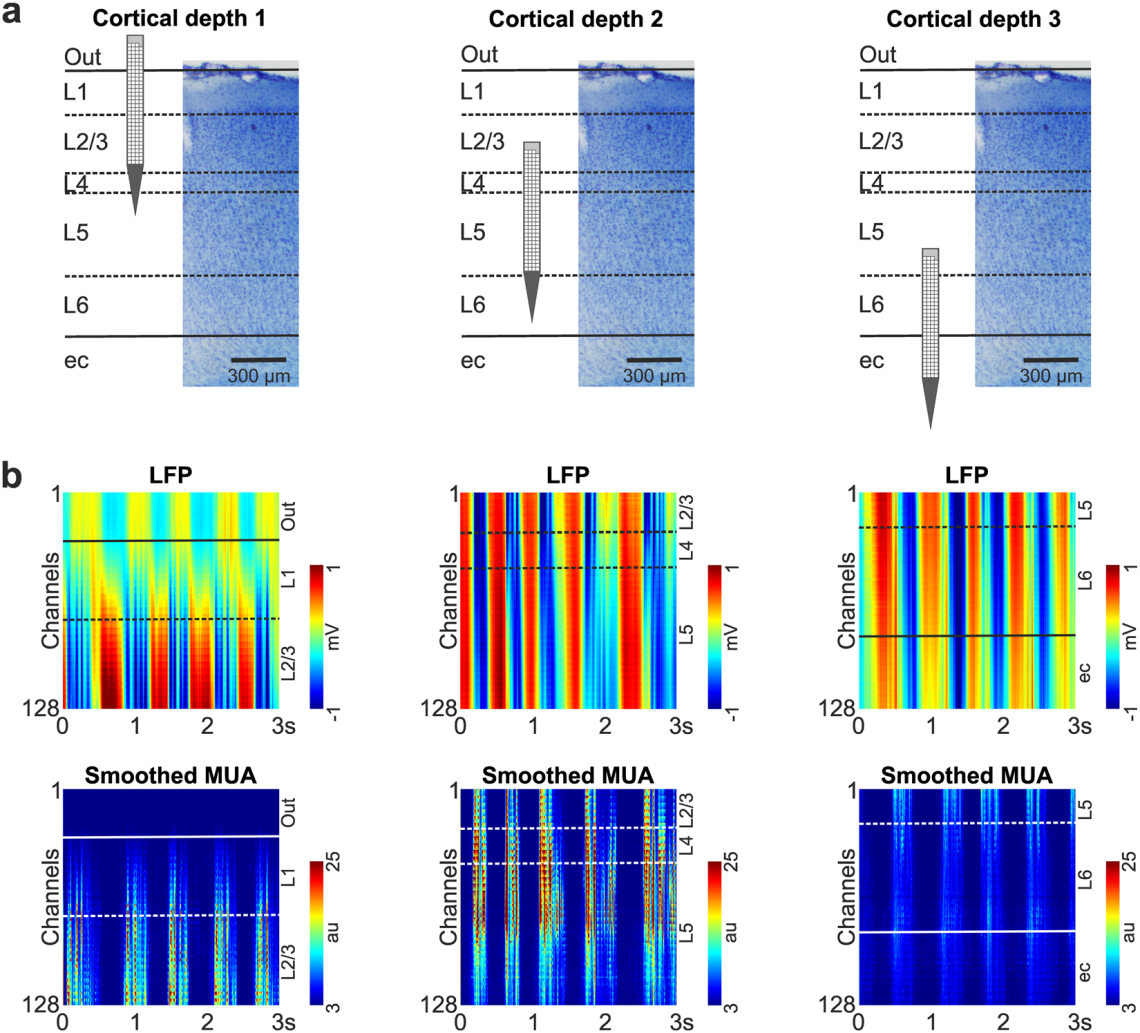
The recording protocol used to collect the cortical data and examples of cortical activity depth profiles. (**a**) The silicon probe was first inserted into the superficial layers of the cortex (left; cortical depth 1; some recording sites at the top of the array were outside of the cortex), then after successful data acquisition it was moved to deeper layers (middle; cortical depth 2). In the case the bottommost sites were still located in the cortex (indicated by the measured activity), the probe was advanced deeper (right; cortical depth 3) to collect activity from all cortical layers. (**b**) Color maps constructed from three-second-long traces of local field potentials (LFP; 0.1–300 Hz; top row) and multi-unit activity (MUA; 300–5000 Hz, rectified; bottom row). All channels were used to construct the color maps. The MUA was smoothed using a 50 Hz lowpass filter. High and low spiking activity on the MUA color maps are indicated by red and blue colors, respectively. Color maps in the same column correspond to the cortical depth shown directly above the maps, on panel a. Estimated positions of cortical layer boundaries are indicated with dashed lines. The dorsoventral (vertical) tissue coverage of the probe is about 717 µm. au, arbitrary unit; ec, external capsule; L, layer.

To verify the recording location of the probe post mortem during the histological processing of the brain tissue^35^, the silicon shank was coated with red-fluorescent dye 1,1-dioctadecyl-3,3,3,3-tetramethylindocarbocyanine perchlorate (DiI, D-282, ~1% in absolute ethanol, Thermo Fisher Scientific, Waltham, MA, USA) before insertion. After that, the silicon probe was mounted on a motorized stereotaxic micromanipulator (Robot Stereotaxic, Neurostar, Tübingen, Germany) and was driven into the brain tissue with a slow insertion speed of 2 μm/sec with the intention to reduce the insertion-related mechanical tissue damage and to increase the single unit yield^28^. During probe insertion, care was taken to avoid rupturing blood vessels located on the brain surface. Neural activity was collected from multiple cortical depths to sample electrical activity from all cortical layers during a single penetration. Because the vertical tissue coverage of the probe used was about 700 μm and the dorsoventral thickness of cortical areas examined was between 1.3 and 1.8 mm (mean ± standard deviation [SD]; 1.51 ± 0.13 mm; measured post-mortem at the probe tracks after histological processing)^26^, we usually recorded from two or three depths, with the recording positions slightly overlapping (from 20 to 100 μm; Fig. 2). Two probe insertions were performed in a single animal (n = 40 penetrations in total), with a distance of at least 500 μm between the insertions to avoid recording from the proximity of brain tissue damaged by the previous insertion. After probe insertion, the brain tissue was allowed to settle for at least 10 minutes before we started recording the cortical activity. A stainless steel needle inserted in the nuchal muscle of the animal served as the reference and ground electrode during recordings. Dehydration of the neocortex was prevented by regularly dropping room temperature physiological saline solution into the cavity of the craniotomy. At the end of the experiment, the silicon probe was retracted and cleaned by immersing it into 1% Tergazyme solution (Sigma-Aldrich, St. Louis, MO, USA) for at least 30 minutes. This was followed by rinsing the probe with distilled water for about 2 minutes.

### Electrophysiological recordings

Spontaneously occurring cortical electrical activity was collected using an Intan RHD-2000 electrophysiological recording system (Intan Technologies, Los Angeles, CA, USA). One 64-channel and two 32-channel headstages were used to record from a total of 128 channels. Wideband signals (0.1–7500 Hz) were acquired with 20 kHz/channel sampling rate and with 16-bit resolution. About 30 minutes of multichannel neuronal data were collected at each recording location (mean ± SD; 37.55 ± 11.29 min; range, 15.13–61.94 min). Data were saved to a local network-attached storage device for offline analysis. A single silicon probe was used during the study and a total of 109 cortical recordings were collected. The recording site – channel mapping is shown in panel a of Fig. 1 (the numbering of channels starts at the site located in the top left corner of the array). The probe used for data acquisition contained two recording sites which were shorted (sites 64 and 65; only in the recordings of Rat04-Rat20), and another site was unfunctional during one of the experiments (Rat20; channel 2). Data on these bad channels might be interpolated using the signal of adjacent channels, however, we did not intend to interpolate or remove these channels here. In the case of one penetration (Rat07; second insertion), no data was recorded because there was visible bleeding during probe insertion which was accompanied by the lack of cortical spiking activity.

### Histology

To detect the track of the silicon probe in the brain tissue and to confirm the recording location, we used a histological procedure described previously^7,28^. Briefly, the animal was deeply anesthetized after the experiment, then perfused transcardially with physiological saline solution (100 ml) followed by a fixative solution containing 4% paraformaldehyde in 0.1 M phosphate buffer (PB, pH = 7.4, 250 ml). The fixed brain was removed from the skull, placed in the fixative solution, and stored at 4 °C. After that, 60-µm-thick coronal brain sections were cut with a vibratome (Leica VT1200, Leica Microsystems, Wetzlar, Germany). The cut sections were washed in 0.1 M PB, then mounted from gelatin onto microscopic slides and air dried. To identify brain sections with fluorescent marks of DiI which indicate the location of the probe track, the slides were examined under a light microscope (Leica DM2500, Leica Microsystems) equipped with a fluorescence LED illumination source (SFL4000, Leica) and with a digital camera (DP73, Olympus, Tokyo, Japan). After that, the brain sections were processed for cresyl violet (Nissl) staining, dehydrated in xylene and coverslipped with DePex (SERVA Electrophoresis, Heidelberg, Germany). Finally, to verify the recording location based on the stereotaxic rat brain atlas^34^ and to identify the borders of cortical layers, Nissl-stained sections containing the track of the silicon probe were photographed under the microscope.

### Estimation of noise and signal levels of recordings

**Fig. 3:**
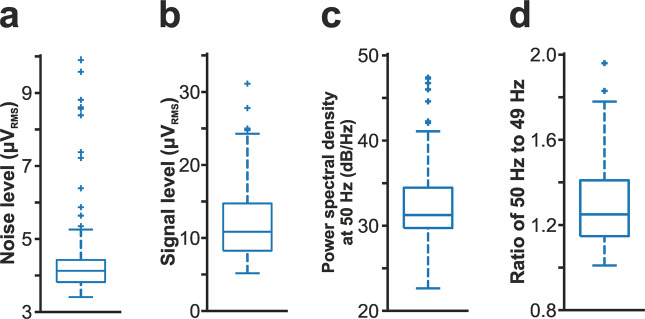
Quality assessment of cortical recordings (n = 109). (**a**) Distribution of the root mean square (RMS) noise levels estimated from the recordings. (**b**) Distribution of the estimated RMS signal levels. (**c**) Distribution of power spectral density values measured at 50 Hz to demonstrate power line noise contamination (see also panel d of Fig. 1). (**d**) Distribution of the ratios of the power spectral density value measured at 50 Hz to the power spectral density value computed at a nearby frequency (49 Hz) without power line noise contamination.

To estimate the noise level in cortical recordings (Fig. 3a), we used a method described previously^23^. In short, we took advantage of the fact that almost all cortical neurons stop firing for a short time during down-states of the ketamine/xylazine-induced slow oscillation (see, for example, in Fig. 1c)^9,26,27^, and used these short time windows of neuronal silence to approximate the level of noise contaminating the recordings. First, a state detection algorithm was used to detect the onset of up- (high spiking activity) and down-states (low spiking activity)^23,26^. After that, on each channel of the filtered (300–6000 Hz; Butterworth 3rd-order bandpass filter; zero-phase shift) and rectified recording, the root mean square (RMS) value of a 50-ms-long segment at the center of down-states with a duration of at least 200 ms was calculated, then the RMS values were averaged between channels located in the cortex. To estimate the signal level in our recordings (Fig. 3b), the RMS of the neuronal activity was calculated in the same way as described above for the RMS noise level, except using measurements during up-states instead of down-states. To assess the power line noise contamination of the dataset (see in Fig. 1d), the power spectral density at 50 Hz was calculated during the first 10 minutes of each recording using SpikeInterface^36^. The power spectral density values of 10 channels located at different cortical depths were averaged. Besides calculating the absolute value of the 50 Hz noise (i.e., the power spectral density value at 50 Hz; Fig. 3c), we also have determined the relative increase in the noise level by calculating the ratio of the power spectral density at 50 Hz to the power spectral density at a nearby frequency value without power line contamination (49 Hz; Fig. 3d). All computed noise and signal values are listed in the „Recording_characteristics” Comma Separated Values (CSV) file available in the dataset.

### Spike sorting

To extract single-unit activity, spike sorting was performed with the Kilosort2 MATLAB package^37^ using the default parameters (available in the StandardConfig.m file) and a channel map file generated based on the recording site layout of the silicon probe used in this study. Only the parameters sampling rate (ops.fs = 20,000), threshold (ops.Th = [8 3]) and minimum firing rate (ops.minfr_goodchannels = 0) were changed. As its output, Kilosort2 generates a list of templates (clusters) which correspond to the single units found in the recording. This list was visually inspected to remove artefactual templates as well as units considered as noise (e.g., units with abnormal spike waveform shapes) or multi-unit activity (e.g., clusters with a contaminated refractory period). Manual curation of the Kilosort2 results was done with the Phy Python library^38^, which provides a graphical user interface for interactive visualization of high-density data and supplies operations for merging, splitting and marking of clusters. In our dataset, we aimed to keep only those clusters (marked as good units) which had at least 100 spikes, a clear refractory period, a trough-to-peak amplitude > 30 µV of the mean spike waveform at the peak waveform channel (for definitions see the next section) and a consistent waveform shape. If two clearly separable clusters were found on any of the two-dimensional scatterplots of principal components calculated from the spike waveforms of a particular unit (FeatureView in Phy), the two clusters were split and the two new clusters were evaluated separately. In some of the cases, two or more clusters were merged if they contained spikes from the same single unit (e.g., in the case of some bursting neurons or when the change in the spike waveform shape due to electrode drift resulted in multiple clusters of the same unit).

After manual curation, a custom MATLAB script was used to detect duplicated units based on their cross-correlograms. A large peak on the cross-correlogram at around zero millisecond was an indicator of putative duplicated clusters. Unit duplication was verified by inspecting the spike waveforms of tagged cluster pairs. In most of the cases the spike waveforms of the two units were temporally shifted with a few samples relative to each other. This was usually caused by Kilosort2 detecting the spikes of units with a large waveform spread on multiple channels located far from each other. In most of the cases the primary cluster could be clearly identified because the negative peak of its spike waveform was centered at 0 ms. All duplicates of the primary cluster were removed. Finally, single units not located in the neocortex (e.g., in some cases hippocampal neurons were recorded at the deepest locations) were removed from the dataset.

As described above in the section detailing the recording protocol, there was some spatial overlap between subsequent recording positions of a particular penetration. Thus, a small fraction of the sorted single units might be included two times in the dataset. However, because the spike waveforms of these units were recorded on different parts of the probe array in the two recordings (and thus they have a different multichannel spike waveform), we did not intend to find and remove these types of duplicated units.

### Extracted single unit properties

**Fig. 4:**
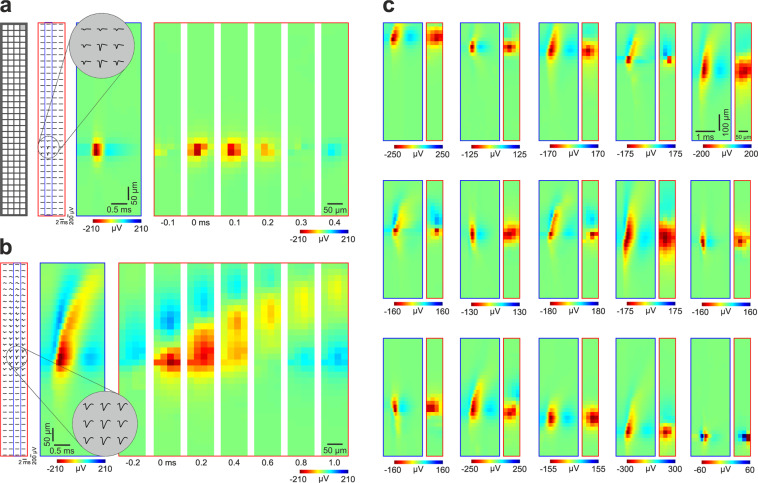
Spatiotemporal profiles of high-resolution spike waveforms of sorted single units. (**a**) Left: schematic of the recording site layout. Middle left: Mean spike waveforms of a cortical neuron with a narrow spike (putative interneuron) on all channels. Middle right: Color map showing the spatiotemporal profile of the spike waveform on channels (n = 32) framed by the blue rectangle in the middle left (column of sites which contain the peak waveform channel). The inset at the top demonstrates magnified mean spike waveforms on nine adjacent channels which recorded the spikes with the largest amplitudes. Right: Color maps illustrating the two-dimensional spatial profiles of the spike waveform on all channels (red rectangle in the middle left) at multiple time points (time step, 0.1 ms). Time zero corresponds to the trough (negative peak) of the spike waveform. (**b**) The spatiotemporal profile of the spike waveform of a neuron with a wide spike (putative principal cell). Note the longer duration and the dorsal propagation of the spike in the color maps (time step on the right, 0.2 ms). (**c**) Additional examples (n = 15) of extracellular spike waveforms of various single units. For each pair of color map, the left (with a blue frame) shows the spatiotemporal pattern of the spike waveform at the column of sites comprising the peak waveform channel. The right color map (with a red frame) illustrates the two-dimensional potential profile of the spike waveform measured at the trough of the waveform. The examples are ordered by the vertical position of their spike waveform on the array. For illustration purposes, only units with their spike waveform located in the center of the array are shown.

**Fig. 5:**
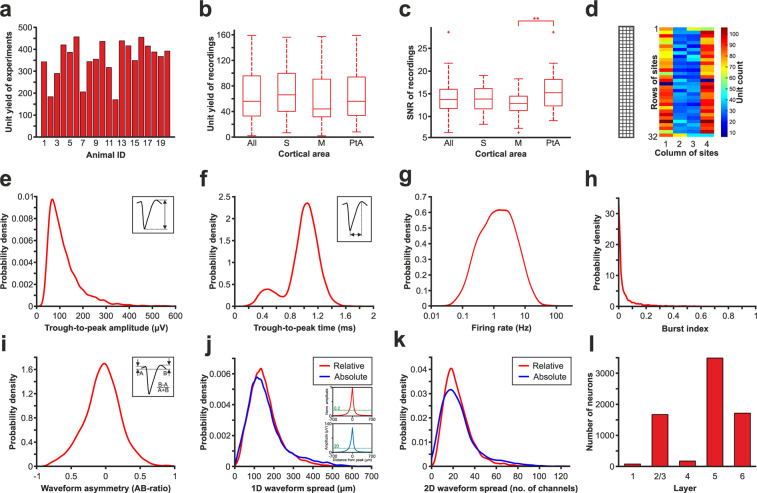
Results of spike sorting and distributions of single unit properties. (**a**) Unit yield of each experiment (n = 20 rats). (**b**) Boxplots showing the distribution of unit yields of recordings (n = 109), also broken down by cortical areas (S, somatosensory; M, motor; PtA, parietal association cortex). (**c**) Boxplots illustrating the distribution of the signal-to-noise ratio (SNR) of recordings estimated by averaging the SNR of single units found in the recordings. **p < 0.01 (one-way ANOVA with Tukey’s honestly significant difference post hoc test). (**d**) Distribution of all single units (n = 7126) based on their soma location (corresponding to the peak waveform channel) plotted according to the recording site layout (on the left). Sites 64 and 65 were shorted (sites with dark blue color in the middle of columns 1 and 4). (**e**–**k**) Probability density functions showing the distribution of single unit properties. (**e**) The distribution of trough-to-peak amplitudes of all single units. The inset shows the calculation method of this property (see also the Methods section for more details). (**f**) Distribution of the trough-to-peak times of all units. This property was defined as the time period between the trough and the subsequent positive peak of the spike waveform (inset). (**g**) Distribution of firing rates. Note that the x-axis is on a logarithmic scale. (**h**) Distribution of burst index values. (**i**) Distribution of AB-ratios showing the spike waveform asymmetry. The inset shows the calculation method of the AB-ratio. (**j**) Distribution of one-dimensional (vertical) relative (red) and absolute (blue) spike waveform spreads of single units. The insets show the average spread of the spike waveforms calculated from normalized (top) and absolute (down) spike amplitudes of all units. The threshold level used to compute the vertical spike waveform spreads are indicated with green dashed lines in the insets. (**k**) Distribution of two-dimensional relative (red) and absolute (blue) spike waveform spreads calculated using the same thresholds as applied for the computation of the vertical spike waveform spreads in panel j. (**l**) Laminar distribution of sorted cortical single units.

Multichannel mean spike waveforms of the selected single units were computed both from the wideband (0.1–7500 Hz) and the filtered (300–6000 Hz bandpass; 3^rd^ order Butterworth filter) continuous recordings (see examples of mean spike waveforms derived from the filtered recordings in Fig. 4). About four millisecond long data snippets (81 sample points in total) were cut from the continuous data on all channels at each spike of a particular unit (with the spike waveform peak located in the center of the snippet), then averaged across spikes. Besides the mean values, the standard deviations were also calculated. Furthermore, for each single unit, we computed and shared the following spike waveform and firing pattern properties. Distributions of these properties are presented in Fig. 5.

The *firing rate* (in Hz) shows the number of spikes fired during one second.

The *burst index* shows the ratio of spikes in bursts to all spikes, as defined in the study of Mizuseki and colleagues^39^. That is, a spike is included in a burst event if the interspike interval between the particular spike and either the previous or the subsequent spike is less than 6 ms.

The *trough-to-peak amplitude* (in µV) shows the absolute amplitude difference between the trough (minimum point) and the peak (maximum point) of the mean spike waveform (computed from the bandpass filtered data) on the channel where the spike amplitude is the largest (*peak waveform channel*; see also in Fig. 5e).

The *trough-to-peak time* (or also referred to as spike duration; in ms) is the time difference between the trough and the subsequent waveform peak of the mean spike waveform (filtered). The spike duration is measured at the peak waveform channel (see also in Fig. 5f).

The *AB-ratio*, which shows the waveform asymmetry, is the ratio between the two positive peaks of the filtered spike waveform measured on the peak waveform channel as defined in the CellExplorer software^40^ (see also in Fig. 5i).

The high spatial resolution provided by the dense sampling of spike waveforms might give additional useful information to characterize neurons. Thus, for each single unit, we extracted the following multichannel waveform features from the mean spike waveforms (computed from the filtered recordings).

#### Relative one-dimensional spike waveform spread (in µm)

This property is defined as the spatial extent of channels on which the trough-to-peak amplitude of the spike waveform exceeds 20% of the amplitude measured on the peak waveform channel. Only those recording sites which were located in the same column of sites as the peak waveform channel were used in this calculation (see the inset in Fig. 5j for the average spike waveform spread calculated from the spike waveforms of all single units). Our calculation methods was similar as described in the study of Jia and colleagues^15^, however, we used a slightly higher threshold (20% instead of 12%). Our intention with the more conservative threshold was to avoid overestimating the waveform spread in certain cases, for example when the background activity was high on several channels due to highly synchronized cortical activity which might affect the mean multichannel spike waveform, or when the signal-to-noise ratio of mean spike waveforms was lower due to low spike counts or low amplitudes.

#### Absolute one-dimensional spike waveform spread (in µm)

The same as above except we used an absolute threshold of 20 µV here.

#### Relative two-dimensional spike waveform spread

This feature is defined as the number of channels on which the trough-to-peak amplitude of the spike waveform exceeds 20% of the amplitude measured on the peak waveform channel. All channels were used in this calculation.

#### Absolute two-dimensional spike waveform spread

The same as above except we used an absolute threshold of 20 µV here.

In most of the cases the trough (negative peak) of the spike waveform of single units was larger compared to their positive peaks. However, a small fraction of single units (~1%) had a positive peak notably larger than their trough. These units, which might be putative axonal spikes^41,42^, were identified and marked in the dataset (*positive spikes*).

### Quality metrics

**Fig. 6:**
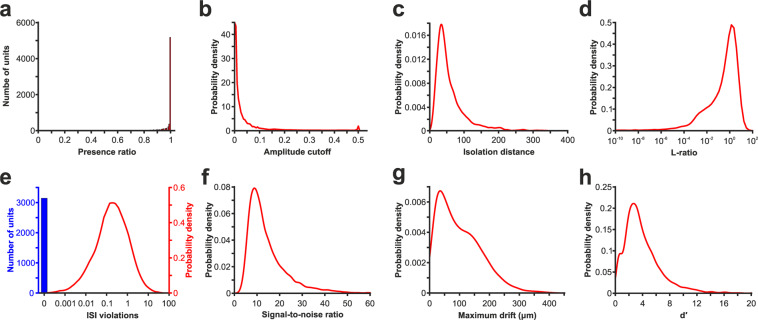
Quality assessment of the spike sorting based on various quality metrics. (**a**) Histogram showing the distribution of the presence ratios. (**b**–**h**) Probability density functions illustrating the distributions of quality metrics. Distribution of (**b**) amplitude cutoffs, (**c**) isolation distances, (**d**) L-ratios (note that the x-axis is plotted on a logarithmic scale), (**e**) interspike interval (ISI) violations (note that because a large fraction of units has an ISI violation of zero, these zero values are demonstrated as a single column (in blue); the height of the column is not proportional to the distribution of other ISI values), (**f**) signal-to-noise ratios, (**g**) maximum drifts, and (**h**) d’ of all single units.

To describe the quality of the selected single units, several quality metrics were calculated and provided with the dataset, along with the properties of single units described above. Quality metrics were computed using the SpikeMetrics module of the SpikeInterface open-source Python framework^36^. Most of these metrics are based on the code developed at the Allen Institute for Brain Science^3^. The complete list and detailed description of the quality metrics can be found on the GitHub page of these software (listed also in the Code Availability section) and in the documentation of the Allen Software Development Kit (https://allensdk.readthedocs.io/en/latest/_static/examples/nb/ecephys_quality_metrics.html), here we provide only a short description. Distributions of some of these metrics are presented in Fig. 6.

#### Presence ratio

Fraction of the recording in which spikes of the unit are present (range, 0–1).

#### Amplitude cutoff

Estimate of the rate of missed spikes (false negative rate) based on the amplitude histogram of spikes (range, 0–0.5).

#### ISI violations

Rate showing refractory-period violations based on the work of Hill *et al*.^43^. Lower values indicate less contamination from the spikes of other single units.

#### Isolation distance

The calculation of this metric is based on the principal components of the spike waveforms and the Mahalanobis distance between spikes^44,45^. It shows the degree of separation of a unit cluster compared to other clusters. A higher value reflects a better isolation.

#### L-ratio

The Mahalanobis distance and chi-squared inverse cumulative distribution function (given the assumption that the spikes in the cluster distribute normally in each dimension) are used to find the probability of cluster membership for each spike^44,46^. Lower values indicate units with better quality.

#### d’

The classification accuracy between single units based on linear discriminant analysis^43^. This feature is also computed in the principal component space.

#### Nearest-neighbors hit rate and miss rate

Non-parametric estimate of unit contamination using nearest-neighbor classification^47^.

#### Silhouette score

A standard metric for quantifying cluster overlap^48^.

#### Maximum drift

Maximum change (in µm) in the position of the spike waveform throughout the recording. This feature can be used to identify recordings with a high amount of probe motion relative to the brain tissue^3^.

#### Cumulative drift

Cumulative change (in µm) in position of the spike waveform throughout the recording^36^.

#### Signal-to-noise ratio (SNR)

Ratio of the spike amplitude and the noise in the recording. The noise level was estimated using the median absolute deviation (MAD) formula implemented in SpikeMetrics.

### Laminar localization of recording sites and single units

For each single unit, we determined the cortical layer where the soma of the neuron was located. We considered the peak waveform channel (i.e., the channel where the amplitude of the spike waveform of the particular unit was the largest) as the soma position of a particular neuron. This is usually a good estimate of the real position of the cell body of neurons^49^. Then, cortical layer boundaries and laminar positions of recording sites were estimated based on the Nissl-stained brain sections and various electrophysiological features were extracted from the cortical local field potentials (LFP; 0.1–300 Hz) and multi-unit activity (MUA; 300–5000 Hz). Using data from all channels, we created LFP and MUA depth profiles of the cortical slow-wave activity from short (few second long) segments of continuous traces (see examples in Fig. 2b). Furthermore, in recordings where a sufficient level of MUA could be recorded, we detected the onset of active phases (phases showing high spiking activity, also called up-states) of the slow-wave activity using a MUA-based state detection algorithm developed in one of our previous studies^26^. Then, we computed the average LFP and MUA depth profiles of these active phases. Combining the anatomical landmarks obtained from the histology with the features extracted from the cortical activity during SWA^26^, we could identify the laminar location of single units with a relatively high reliability. However, some uncertainty may remain in the case of units located near the border of layers or, due to slight brain dimpling, in the case of units located in superficial layers. The laminar location of units and recording sites is also provided in the dataset.

## Data Records

The dataset and corresponding metadata are publicly available via the G-Node Infrastructure (GIN, https://gin.g-node.org/) provided by the German Neuroinformatics Node of the International Neuroinformatics Coordination Facility (https://gin.g-node.org/UlbertLab/High_Resolution_Cortical_Spikes)^50^. Each recording and corresponding metadata, single unit properties and quality metrics were packaged in the Neurodata Without Borders: Neurophysiology version 2.0 (NWB:N 2.0) data format^20^ with custom MATLAB scripts using the MatNWB application programming interface (API). A single NWB file was created from each recording (n = 109). NWB files were placed in the appropriate folder based on the identifier of the animal (from Rat01 to Rat20), insertion sequence (Insertion1 or Insertion2) and cortical depth (from Depth1 to Depth3). The filename of the NWB file (identifier) was constructed by concatenating the above information (e.g., Rat01_Insertion2_Depth3).

The NWB file, which, in terms of storage, is based on the Hierarchical Data Format (HDF5), contains several main groups which are similar to directories. The acquisition group contains the continuous wideband 128-channel data (‘wideband_multichannel_recording’) in a compressed form, as well as several parameters related to the raw data such as the measurement unit or the data conversion number. The general group contains metadata about the experiments and consists of several subgroups, related to the recording probe (‘general/devices’; ‘general/extracellular_ephys’) or the subjects of the experiments (‘general/subject’). Former subgroups carry information about the probe location (brain area and stereotaxic coordinates) and the relative positions and laminar location of recordings sites, while the latter contains metadata about the animal (e.g., sex, species, subject ID, or weight). Information about spike sorting and single units and corresponding data are available in the units group. For each unit, we included here the mean and standard deviation of their spike waveform on all channels, calculated both from the filtered (‘mean_waveform_all_channels_filt’; ‘waveform_sd_all_channels_filt’) and the wideband data (‘mean_waveform_all_channels_raw’; ‘waveform_sd_all_channels_raw’). For an easier visualization of the spike waveform in two dimensions, we have also added an array which contains the mean spike waveform in the 32 × 4 shape of the array (‘mean_waveform_all_channels_filt_32 × 4’; ‘mean_waveform_all_channels_raw_32 × 4’). Furthermore, the spike waveform recorded on the channel with the largest spike (i.e., peak waveform channel) was saved separately (‘mean_waveform_peak_channel_filt’, ‘mean_waveform_peak_channel_raw’). All single unit properties and cluster quality metrics described in the Methods section, as well as the spike times and spike count of each unit were saved in the units group. However, if needed, potential future users can use the provided wideband recordings to redo the spike sorting either by applying different parameters in Kilosort2 or using other spike sorting algorithms. Furthermore, to aid users in selecting and analyzing a subset of this dataset appropriate for their research goals, we also created an NWB file (‘allSingleUnits.nwb’) which contains all single units with all the properties listed above, along with the identifier of the recording (‘units/session_id’) and the cortical area (‘units/cortical_area’) they originate from.

Besides the NWB files, two CSV files are included in the dataset. The first (‘Animal_characteristics_and_targeted_cortical_areas’) contains the information shown in Table 1, while the second CSV file (‘Recording_characteristics’) lists several useful properties for each NWB file including the file size, the duration of the recording, the cortical area, the single unit yield, the average signal-to-noise ratio of single units, the degree of power line (50 Hz) noise contamination, as well as the RMS noise and RMS signal levels.

## Technical Validation

The cortical dataset (n = 109 recordings; ~0.9 TB overall size) was collected using a single-shank silicon-based multielectrode providing dense sampling of brain electrical activity (Fig. 1a). Rats were anesthetized with ketamine/xylazine cocktail which is known to induce slow-wave activity (SWA) in the thalamocortical system of rodents (Fig. 1b,c)^26,27,51,52^. During SWA, the neocortex shows characteristic patterns of electrical activity, for example, alternating phases of high and low spiking activity (Fig. 1c). The peak frequency of the ketamine/xylazine-induced SWA is usually between 1 and 2 Hz in rats^26,53^ (Fig. 1d). In our dataset, we measured an average peak frequency of 1.29 ± 0.44 Hz suggesting an ordinary appearance of SWA (Fig. 1e). The fluorescent marks left in the brain tissue by the dye painted on the probe were used to identify the cortical recording location, that is, the tracks caused by the two probe insertions in the cortex (Fig. 1f). After we have found the brain sections containing these fluorescent marks, anatomical features provided by Nissl-staining were used to identify the boundaries of cortical layers and to determine the laminar location of recording sites (Fig. 1f). To sample data from all layers, cortical electrical activity was recorded in most cases from two to three depths (depending on the thickness of the cortex; Fig. 2). The laminar position of the soma of single units was estimated using the combination of anatomical (e.g., layer borders) and electrophysiological features (local field potential and multi-unit activity depth profiles^26^; Fig. 2). Estimated noise and signal levels of the cortical recordings are shown in Fig. 3 (see the Methods section on the detailed procedures of noise and signal level calculation). The quality of most of the recordings was high, with high signal (spiking activity) and low noise levels. Only a few measurements (~10) were contaminated with higher levels of noise.

Single-unit activity was extracted from the continuous wideband data using spike sorting, then various spatial and temporal features of the single units were calculated to characterize our dataset and to assess its quality (Figs. 4–6). The neocortex is composed of two main neuron types, inhibitory (GABAergic) interneurons and excitatory principal cells. These two neuron classes can be discriminated based on the durations of their extracellular action potentials: interneurons generate usually narrow spikes, while most principal cell fire wide spikes^54^. To demonstrate example cortical single units from our dataset, in Fig. 4 we show the high-resolution spike waveforms of a neuron with a narrow spike (Fig. 4a), a putative principal cell firing wide spikes (Fig. 4b), as well as a small collection of units with various spike waveforms (Fig. 4c). The spike waveform of the putative principal cell shows also a propagating pattern indicating the backpropagation of the action potential from the soma to the apical dendritic tree (Fig. 4b). This feature is characteristic of pyramidal cells and was observed in previous studies^15,55^.

In total, we sorted more than 7000 single units, with around 2300 units from each of the three main cortical areas (Table 2). On average, about 350 neurons could be sorted from the data obtained in a single rat (Fig. 5a; Table 2) and 180 units from a single penetration (Table 2). From a single recording acquired at a particular cortical depth, we could extract around 65 neurons on average (range: 2–159) and there was no statistically significant difference in the unit yield between the three cortical areas (one-way ANOVA, F(2, 108) = 1.27, p = 0.286; Fig. 5b; Table 2). The quality of recordings was also estimated by averaging the SNR of neurons sorted from these recordings (Fig. 5c). Interestingly, we found a statistically significant difference between the SNR values of cortical areas (one-way ANOVA, F(2, 108) = 5.99, p = 0.0034). Tukey’s honestly significant difference post hoc test revealed that the quality of data acquired from the parietal association cortex was significantly better than the quality of motor cortical recordings (p = 0.0022). The cause behind this difference in quality is most likely the sequence of probe insertion. We have found a significant difference in the RMS signal levels (Wilcoxon Rank Sum Test; p = 0.00034) and also in the SNR values (two-sample t-test; p = 0.0066) between recordings obtained after the first and the second probe insertion. Both RMS signal levels (mean ± SD: 13.56 ± 5.23 µV_RMS_ vs. 10.41 ± 4.23 µV_RMS_) and SNR values (14.79 ± 3.22 vs. 12.98 ± 3.54) were significantly higher after the first insertion compared to the second probe penetration. Furthermore, more single units were sorted from the data obtained after the first insertion (4377 units vs. 2749 units) and the amplitudes of these units also were significantly higher (Wilcoxon Rank Sum Test, p = 3.51 × 10^−30^; mean ± SD: 128.07 ± 85.84 µV vs. 108.82 ± 68.76 µV). Recordings from the motor cortex were obtained in more cases after inserting the probes the second time (n = 14; 70% of the second insertions) compared to data acquired from the parietal association and somatosensory cortices (n = 4 and n = 1 second insertions, respectively).

**Table 2:** Single unit yield statistics (average ± standard deviation) calculated for brain regions, insertions and recordings.

	All	S	M	PtA
Total unit yield	7126	2518	2362	2249
Average unit yield per animal	356.45 ± 86.06	—	—	—
Number of penetrations	39	12	15	12
Average unit yield per penetration	182.79 ± 65.13	209.83 ± 56.57	157.47 ± 59.19	187.42 ± 72.79
Number of recordings	109	35	41	33
Average unit yield per recording	65.40 ± 40.98	71.94 ± 40.71	57.61 ± 39.17	68.15 ± 43.11

We also calculated the distribution of single units at the recording sites of the array by assigning each unit to the site corresponding to its peak waveform channel (Fig. 5d). Here we can see that most units were detected near sites located closer to the edge of the silicon shank. The reason behind this, about twofold difference is that besides neurons located in front of edge sites, cells located next to edge sites (in the cortical tissue next to the probe shank) were assigned to these sites, while only those neurons which are in front of center sites were assigned to center sites.

We also computed several spatial and temporal attributes of the mean spike waveform, as well as properties related to the firing patterns of single units (Fig. 5e–k). The mean amplitude of the spike waveforms calculated from the filtered (300–6000 Hz) recordings was 120.64 ± 80.23 µV (Fig. 5e), while the spike durations showed the typical bimodal distribution (Hartigan’s dip test; p = 0.023) characteristic of the neocortex with shorter durations corresponding to putative interneurons and longer durations to putative principal cells (Fig. 5f). The firing rate of units showed a lognormal distribution, as described previously (Fig. 5g)^56^. High-resolution recordings also allow to examine the spatial spread of the spike waveforms. On average, the vertical spread of spike waveforms was about 160 µm (Fig. 5j), while, considering the whole, two-dimensional array, spike waveforms could be recorded with around 25 recording sites simultaneously (Fig. 5k). Based on the laminar location of the single units, almost half of the recorded neurons were located in layer 5, while around the quarter of all neurons originated from layer 2/3 and another quarter from layer 6 (Fig. 5l). Only a small fraction of units were located in layers 1 and 4. This is in agreement with the findings of previous studies showing that in the neocortex the strongest activity during SWA can be recorded in layer 5, while the unit activity decreases both above and below this layer^9,26,52^.

Finally, we provide here the results of several spike sorting quality metrics (Fig. 6). The distribution of the values of most of these metrics was similar to metrics calculated from a large dataset of single units collected in a recent study with Neuropixels probes from mice^3^. We also support their approach for single unit inclusion described in the study, that is, keeping all units with an appropriate quality and then, based on the calculated quality metrics as well as on spike waveform properties demonstrated above, future users can filter the collection of single units and select a subset of these based on their research goals.

## Usage Notes

Users can import data from NWB files using the PyNWB and MatNWB APIs^20,22^, or using SpikeInterface^36^. Loaded samples of the raw data have to be multiplied by a conversion number (0.195) to get the amplitudes in microvolts. The recording site – channel mapping is shown in panel a of Fig. 1. Here we provide some examples how users can import data from NWB files using the MatNWB API.

Loading a short segment (20.000 samples corresponding to 1 second of data) of the raw wideband recording on all (128) channels:

1. nwb = nwbRead(‘Rat01_Insertion1_Depth1.nwb’);
2. dataChunk = nwb.acquisition.get(‘wideband_multichannel_recording’).data.load([1, 1], [128, 20000]);

It is important to note that TimeSeries data types in NWB files are stored with time in the first dimension and channels in the second, but dimensions are reversed in MatNWB.

Loading and plotting the mean spike waveform of a specific single unit on the peak waveform channel:

3. peakChannels = nwb.units.vectordata.get(‘peak_waveform_channel’).data.load();
4. meanWaveforms = nwb.units.vectordata.get(‘mean_waveform_all_channels_filt’).data.load();
5. mySingleUnit = 11;
6. singleUnitWaveform = meanWaveforms(peakChannels(mySingleUnit),:, mySingleUnit);
7. plot(singleUnitWaveform);

Loading the isolation distance quality metric of all units found in a single NWB file:

8. IDvalues = nwb.units.vectordata.get(‘isolation_distance’).data.load();

Spike times are stored in a special structure called ragged arrays consisting of two vectors^20^. The spike_times vector contains all spike times of all single units concatenated one after the other, while the spike_times_index vector stores where the spike times of individual single units are located in the spike_index vector. We can load the spikes times of a specific single unit (in seconds) the following way:

9. allSpikeTimes = nwb.units.spike_times.data.load();
10. spikeTimesIndex = nwb.units.spike_times_index.data.load();
11. spikesOfSingleUnit2 = allSpikeTimes(spikeTimesIndex(1) + 1: spikeTimesIndex(2));

SpikeInterface can also be used to load the wideband data and single unit properties:

1. import spikeextractors as se
2. nwbPath = ‘Rat01_Insertion1_Depth1.nwb’
3. recording = se.NwbRecordingExtractor(nwbPath)
4. sorting = se.NwbSortingExtractor(nwbPath)
5. mySingleUnit = 2
6. sorting.get_unit_property(mySingleUnit, ‘isolation_distance’)

## Data Availability

All software used for the visualization, processing and analysis of this dataset are open access or custom written, Python- and MATLAB-based programs. The Kilosort2 MATLAB package was used for spike sorting (https://github.com/MouseLand/Kilosort; version 2.0; commit date, 8 April 2019)^37^, and the Phy Python module for subsequent manual curation of the data (https://github.com/cortex-lab/phy; version 2.0b1; release date, 7 February 2020). Spatial and temporal features of the single unit spike waveforms were calculated using custom MATLAB scripts or the CellExplorer MATLAB module (https://cellexplorer.org/; https://github.com/petersenpeter/CellExplorer; version 1.2; commit date, 25 September 2020)^40^. Single unit quality metrics were computed using the SpikeMetrics module (https://github.com/SpikeInterface/spikemetrics; version 0.2.2; commit date, 30 December 2020) of the SpikeInterface Python-based framework (https://github.com/SpikeInterface/spikeinterface; version 0.11.0; commit date, 10 December 2020)^36^. SpikeMetrics relies on the code of the quality metrics module developed at the Allen Institute for Brain Science (https://github.com/AllenInstitute/ecephys_spike_sorting/tree/master/ecephys_spike_sorting/modules/quality_metrics)^3^. NWB files were created using the MatNWB (https://github.com/NeurodataWithoutBorders/matnwb; version 2.2.4.0; commit data, 9 February 2021) application programming interface.
